# Do Climate Change Policies Promote or Conflict with Subjective Wellbeing: A Case Study of Suzhou, China

**DOI:** 10.3390/ijerph13030344

**Published:** 2016-03-21

**Authors:** Miaomiao Liu, Yining Huang, Rosemary Hiscock, Qin Li, Jun Bi, Patrick L. Kinney, Clive E. Sabel

**Affiliations:** 1 State Key Laboratory of Pollution Control and Resource Reuse, School of the Environment, Nanjing University, 163 Xianlin Avenue, Nanjing 210023, China; memo0904@sina.com (M.L.); ning.rossy@hotmail.com (Y.H.); 2 School of Geographical Sciences, University of Bristol, Bristol BS8 1SS, UK; r.hiscock@bristol.ac.uk (R.H.); c.sabel@bristol.ac.uk (C.E.S.); 3 Suzhou Environmental Science Research Institute, Suzhou 215007, China; qinli_sz@163.com; 4 Collaborative Innovation Center of Atmospheric Environment and Equipment Technology, Nanjing 210023, China; 5 Mailman School of Public Health, Columbia University, New York, NY 10023, USA; plk3@cumc.columbia.edu

**Keywords:** wellbeing, climate change, co-benefits, policy implications, Chinese city

## Abstract

As public expectations for health rise, health measurements broaden from a focus on death, disease, and disability to wellbeing. However, wellbeing hasn’t been incorporated into the framework of climate change policy decision-making in Chinese cities. Based on survey data (*n* = 763) from Suzhou, this study used Generalized Estimation Equation approach to model external conditions associated with wellbeing. Then, semi-quantitative analyses were conducted to provide a first indication to whether local climate change policies promote or conflict with wellbeing through altering these conditions. Our findings suggested: (*i*) Socio-demographic (age, job satisfaction, health), psychosocial (satisfaction with social life, ontological security/resilience) and environmental conditions (distance to busy road, noise annoyance and range hoods in the kitchen) were significantly associated with wellbeing; (*ii*) None of existing climate change strategies in Suzhou conflict with wellbeing. Three mitigation policies (promotion of tertiary and high–tech industry, increased renewable energy in buildings, and restrictions on car use) and one adaption policy (increasing resilience) brought positive co–benefits for wellbeing, through the availability of high-satisfied jobs, reduced dependence on range hoods, noise reduction, and valuing citizens, respectively. This study also provided implications for other similar Chinese cities that potential consequences of climate change interventions for wellbeing should be considered.

## 1. Introduction

Strategies to stabilize the climate are likely to have a wide range of ancillary effects that are not directly related to carbon emissions [1]. The most studied of these are consequences for physical health (diseases and mortality), which has been somewhat incorporated into the framework of policy making [2]. However, as public expectations for health broaden from death, disease, and disability towards wellbeing, policy makers are beginning to consider whether climate change policies promote or conflict with wellbeing [3]. To answer the questions, we have to know what kinds of external conditions, including socio–demographic, psychosocial and environmental factors, are the important determinants of wellbeing and how they are being changed by climate change policies.

Previous studies have examined the relationship between wellbeing and external conditions that might be altered by climate change policies [4]. However, definitive answers to this question have not yet been made due to the following limitations. Firstly, the definition of wellbeing remained open to considerable debate [4,5], which allowed researchers to use wellbeing measurements for their own purposes. Various wellbeing measurements adopted in previous empirical studies [6,7,8,9,10] made it less possible to tell the extent to which a change of wellbeing on a particular scale could be generalized to another. The difficulties in generalizing these results are raising calls to choose widely-used wellbeing measurements in policy analysis. Secondly, existing studies usually focused on depicting the associations of wellbeing with just one or one kind of determinant rather than taking an overview [11,12,13,14,15,16,17,18,19]. Thirdly, studies incorporating wellbeing within the impact assessment of climate change related policies are still scarce [3,4,8,20,21,22,23,24]. Milner *et al.*, explored the impacts of housing energy efficiency on thermal comfort, and associated wellbeing [20]. Nazelle *et al.* noted that promoting active travel improved mental health through walkable neighborhoods [21]. Younger *et al.* suggested that policies aimed at reducing motor vehicle use might have the co-benefit of increasing physical activity and wellbeing [22]. Braubach *et al.* looked at the impact of reducing motor vehicle use, promoting electric cars and building underground railways on wellbeing via noise declines [8]. Van Kamp *et al.* examined the wellbeing effects of adaptation measures, e.g., cooling systems, mechanic ventilation systems and wind turbines parks via altering low frequency noise annoyance [23]. It can be seen that these scarce studies usually focused on the wellbeing impact of single-sector policy rather than comprehensive policy package. And these scarce studies usually focused on the wellbeing impact of single–sector policy rather than comprehensive policy package, which could not give us an overview of integrated interventions’ effects in a city. Additionally, it is important to acknowledge the local context given the variations found in the wellbeing scores in different settings and cultures [4]. Evidences were mostly from developed countries, so more studies are needed in developing countries to understand theoretical and empirical links behind such countries’ policies’ consequences for wellbeing.

This study aimed to extend the existing literature by examining the case of Suzhou, China. As one of the most developed cities in east China, Suzhou is faced with particular challenges from climate change. Specifically, a warmer atmosphere means more extreme summer heat and more deaths in heat waves. Higher temperatures also speed up chemical reactions that form ground-level secondary air pollutants-well-known lung irritant and asthma triggers. Moreover, more hot weather, flooding and other extreme weather events put heavy burdens on infrastructures like electrical supplies, the drinking water system, and the transport system. In the long run, this shift in temperature and water patterns will alter natural habitats through, for example sea level rise and cause huge ecological and economic crises. To address these challenges, Suzhou has been motivated to mitigate and adapt to climate change and has been approved by National Development and Reform Commission as one of the second batch of “low carbon development pilot cities” and consequently agreed to reach its peak of total carbon emissions by 2020 [25]. However, Suzhou is now experiencing serious hot-spot effects of energy consumptions and greenhouse gas emissions. To deal with the tension between high energy demands and ambitious carbon emission control targets in the future, tough climate change mitigation and adaptation policies are expected to be implemented in Suzhou. Table 1 shows the list of these two sets of policies summarized from officially approved “Low carbon development planning of Suzhou (2011–2020)” [25]. On the basis of literature review, it could be seen that climate change interventions in Suzhou will significantly alter external conditions, such as socio–demographic (employment, health), psychosocial (ontological security/resilience), and environmental (noise, air quality) conditions (Table 1). Some critics proposed that through altering these external conditions, some climate change interventions would have negative effects on wellbeing. For example, restrictions on private cars limit individual freedom to buy or use a car, which could make commuting inconvenient and uncomfortable [26]. However, until now it is still a subjective judgment rather than a data-based conclusion as evidences for the nexus between interventions and wellbeing were so scarce. Therefore, there is an urgent need to understand whether climate change policies in Suzhou promote or conflict with wellbeing. More importantly, as a leading city, Suzhou’s mode of development is of interest to an increasing number of Chinese cities. The situations that Suzhou is faced with today will be that other cities have to deal with in the future. So findings in Suzhou have wider implications.

By examining whether climate change policies in Suzhou promote or conflict with wellbeing, this study will contribute to the existing literature in three ways. Firstly, it explicitly links wellbeing to climate change policies via relationships with external conditions that affect wellbeing and are themselves impacted by policy change. Secondly, unlike previous studies aimed at single-sector policy assessment, a climate change policy package including industry policies, transport policies, building policies and others has been examined in this study. Thirdly, the use of data from a structured questionnaire survey in Suzhou contributes to the literature on wellbeing from the perspective of a developing country.

## 2. Methodology and Data

Two steps have been conducted to examine whether climate change policies in Suzhou promote or conflict with wellbeing. Firstly, based on a questionnaire survey in Suzhou, we established a regression model to depict the associations between self-reported subjective wellbeing and external conditions using Generalized Estimation Equation approach. Secondly, we examined whether the associations between these external conditions and wellbeing were likely to imply that a number of greenhouse gas policies were beneficial or detrimental to wellbeing.

### 2.1. Wellbeing Regression Model

#### 2.1.1. Data Collection

To obtain data on wellbeing and its potential determinants, a questionnaire survey (appendix I in Supplementary Information) was conducted in Suzhou in August 2013 with the help of about 40 volunteers. To guarantee the spatial representatives of the samples, we distributed the questionnaires at eight sites located in different districts of Suzhou with a large pedestrian volume (Table S1). We adopted the following measures to guarantee the data quality of our survey. Firstly, we conducted a small preliminary survey in April of that year. Based on the feedback, we adopted a more structured format. Secondly, we trained our volunteers before the survey to make sure that they could answer respondents’ questions about our survey. We were advised by the Nanjing University ethics committee that ethical permission was not needed but informed consent should be obtained. Thus a panel in the front page of our paper questionnaire, elaborated the purpose and content of the survey (see appendix I in Supplementary Information). Before participants filled the questionnaire, our volunteers asked them to read the panel carefully and indicated that their answers could be used for research purposes. Moreover, the data has been anonymized and analyzed at group level in this research, which maximized the protection of personal privacy.

#### 2.1.2. Choice of Wellbeing Measures and Potential Determinants

The WHO–5 wellbeing scale was applied to measure subjective wellbeing (available from: http://www.who–5.org/) in this study for following reasons. Firstly, only five items were included in WHO–5 scale to measure wellbeing over the last two weeks: “I have felt cheerful and in good spirits”, “I have felt calm and relaxed”, “I have felt active and vigorous”, “I woke up feeling fresh and rested” and “My daily life has been filled with things that interest me”. This short scale reduced respondent burden, but still measured hedonic (emotions, the first four items) and eudemonic (meaning, the last item) aspects of wellbeing. Moreover, it has been translated into many languages and successfully statistically validated in a variety of populations [41,42,43,44,45]. More importantly, it was included within the European Quality of Life Survey (EQLS), which made it possible to compare this study with international studies in the future. There were six response options: “all of the time”, “most of the time”, “more than half the time”, “some of the time” and “at no time”. The most positive response scored 5 and the least positive scored 0. Total scores were created by summing scores from each item and multiplying by 4 so that a total score of 0 indicated the lowest wellbeing and a total score of 100 represented the highest wellbeing. Total wellbeing score was the dependent variable in the regression model. 

Based on existing literature, 38 variables associated with wellbeing were chosen as independent variables and divided into six groups: personal, health, satisfaction, ontological security, housing environment and others (Table 2). Age was found not to have a linear relationship with wellbeing so it was analyzed as a categorical variable with four bins: ≤22, 23–39, 40–59, and ≥60. Gender was also analyzed as a binary variable. Regarding socioeconomic status (SES), three bins (low, middle and high) were generated according to the following rules: low SES was signaled by having a basic level of education (middle school education or less) and/or being unemployed and/or having a low household income (less than 5000 CNY per month) and not having any of the indicators of high SES. The indicators of high SES were living in an owner occupied apartment/house and/or having a high annual household income (more than 30,000 CNY per month) and not having any of the indicators of low socioeconomic status. All other respondents were categorized as middle SES.

Health indicators were measured as a dichotomy such as with physical medical condition *vs.* no condition, depressed *vs.* not depressed, and smoker *vs.* non–smoker. Satisfaction with different domains of life was measured on a 5 point Likert scale with responses ranging from highly unsatisfied to highly satisfied. Population resilience was seen through the concept of ontological security which implicitly assumed that wellbeing was affected by outside, and therefore modifiable, sources rather than fixed personality traits. It has been defined as: “The confidence that most human beings have in the continuity of their self-identity and in the constancy of their social and material environments. Basic to a feeling of ontological security is a sense of the reliability of persons and things” [46]. Resilience can be developed through three components: protection from harm, control to make decisions and to be viewed in a positive way by other people or prestige. Development of these three domains enables people to be able to cope with changing circumstances [47]. Ontological security was measured through a modified form of the validated psychosocial benefits from home scale to apply to wider aspects of people’s lives [47] which was used in a study of smokers attempting to cope with the challenge of quitting smoking [48]. The ten items are intended to measure psychosocial feelings which may connect wellbeing to external factors (Table 3). All items are answered on a 5 point Likert scale with responses ranging from strongly agree to strongly disagree.

For housing environmental group, seven variables were considered in this study based on both theory and empirical evidence (Table 2). As proposed by Ulrich’s stress reduction theory, humans have a genetic inclination to respond positively to environments favorable to survival and safety [49]. Exposure to settings that contain nature like green space helps people recover more quickly from the psychological symptoms of stress, leading to reduced negative effect [9,10,49]. In this study, exposure to nature was measured as a categorical variable of “distance to green space” with three bins: less than 10-min walk, 10 to 30-min walk, and more than 30-min walk.

Studies also showed that exposure to community noise caused reactions such as increases in blood pressure, heart rate and finger pulse amplitude, cardiac arrhythmia and changes in respiration and body movements and then led to the reduction in perceived sleep quality, increased fatigue, decreased mood or wellbeing [50,51]. So noise exposure was measured through two categorical variables. The first is a variable of “frequency of being annoyed by noise” with three bins: at no time, less than half of the time, and more than half of the time; the second is that respondents were asked whether they lived “near to busy road” with the response measured as a dichotomy (Yes *vs.* No). Note that in addition to noise exposure, the second variable may also measure easier access to facilities like bus stops, supermarkets, and pharmacies, which could lead to increased wellbeing. In this study, we included this variable to explore the tradeoffs, which have not been clarified in previous studies. 

The variables “the use of heating/cooling”, “main household fuel type”, and “the use of range hood in the kitchen” were indicators of housing quality, such as indoor temperature and air quality, which were proved to significantly change wellbeing [52,53]. We measured them as categorical variables with several bins (see more details in Table 2). In addition to the physical housing environment, humanistic elements like social connections embedded in housing environment also significantly change wellbeing [54,55]. We measured this using a categorical variable of “frequency of exchanging small favors with neighbor” with four bins: Never, Once a week, 2–4 time a week, more than 4 times a week. 

We included seven variables measuring environmental opinions answered on a 5 point Likert scale with responses ranging from strongly agree to strongly disagree. People’s responses reflected important dimensions of their risk perceptions of environmental problems, which have been proved to be an important determinant of happiness [56]. Previous studies also pointed out that psychosocial benefits were gained from private motor vehicle transport compared to public transport [26]. Thus respondents’ main transport mode was measured as categorical variable with six bins.

#### 2.1.3. Statistical Analysis

SPSS version 21 (IBM, Beijing, China) was used to analyze the relationship between wellbeing and these factors. Firstly, univariate analysis was conducted in order to understand variable distributions. Significant skew was said to be presented if skew > (2* standard error of skew). Secondly, bivariate analysis was conducted between wellbeing and each of the other variables. Significance was tested using the Mann Whitney U test for independent variables with 2 categories and the Kruskall Wallis test where there were more than 2 categories and Spearman’s Rho for continuous independent variables. Some variables that were not significant or were thought to have drawbacks were excluded from further modelling at this stage. Thirdly, multivariate analysis proceeded as follows. (1) The base model was constructed, which only included three variables (age, gender and SES). Age, gender and SES were included in models to take into account differences between the survey sample and the population; they are referred to as “design variables”; (2) Each variable was added separately to the base model and then removed and the next variable were entered; (3) Variables of similar groups were added together to the base model and then removed and the next group of variables were entered. On some occasions the standard error of a variable increased by more than 50% compared with the base model. This indicated multicollinearity. Variables were removed until no variables standard error increased by more than 50%. After eliminating the multicollinearity, non–significant variables (*p* < 0.10) were also removed; (4) The satisfaction and ontological security variables were added together and multicollinearity was eliminated as previously. Non–significant predictors (*p* < 0.10) were removed; (5) All remaining variables (where *p* < 0.10 in previous models) were entered simultaneously. Multicollinearity was eliminated as previously. Non–significant predictors (*p* < 0.10) were removed; (6) Variables that had previously become non–significant were added individually unless they were thought to be likely to cause multicollinearity; (7) As variables were added from sixth step, this process was repeated but variables were only added if *p* < 0.05. (8) Variables were removed from the model until all variables in the model were *p* < 0.05. Multicollinearity was tested as previously.

### 2.2. Wellbeing Impact Assessment

As a first step to incorporate wellbeing into the decision process of climate change policy, we matched external conditions altered by climate change policies with those associated with wellbeing in Suzhou. Following this semi-quantitative analysis, we can make suggestions about whether a policy is more likely to enhance or reduce wellbeing.

## 3. Results

### 3.1. Descriptive Statistics

In eight locations of Suzhou, residents were asked to take part in the survey and a 100% response rate was achieved (*n* = 775). About 70% of questionnaires had no missing data. Compared with Suzhou’s population, survey respondents were more likely to be female, younger, and more educated (Table S2). This response pattern has also been found in previous social studies [57]. Overall, the sampling biases indicated by these differences were small but significant, so all multivariate models included age, gender and SES, which were known as “base model”.

Tables S3–S9 showed the descriptive statistics of independent variables. The descriptive statistics of the WHO–5 wellbeing scale were shown in Table 4. Respondents’ scores ranged from 0 to 100 and the interquartile range was 44 to 68. The mean was 55 and the median was 56. The wellbeing scale was significantly negatively skewed. This meant that medians rather than means needed to be calculated in bivariate analysis and the scale was transformed for multivariate analysis.

### 3.2. Bivariate and Multivariate Analysis

Tables S3–S9 also showed the results of bivariate analysis between wellbeing and variables in each group, respectively. Most variables were significantly related to wellbeing (*p* < 0.05) at this stage with the exception of household fuel type, frequency of exchanging small favors with neighbors, traffic mode, smoking and three environmental opinion related variables. These seven variables were excluded from further modelling at this stage. The multivariate modelling was presented in Tables S3–S9 for steps 1 to 3 and the final model was presented in Table 5.

The only design variable that was significant in the final model was age. Respondents under 23 years old had significantly higher wellbeing than those aged 40–59 suggesting that the youngest respondents had wellbeing scores that were 8 points higher than those aged 40 to 59. Of the seven satisfaction variables tested, satisfaction with job, satisfaction with health and social life were significantly associated with wellbeing. An increase of one point on a 5-point rating scale of satisfaction with job, health and social life appeared to correspond on average to a 2.2%, 2.8% and 4.7% increase of the wellbeing scale, respectively. Of the ten ontological security variables tested, “other people would like a life like mine”, “I feel safe”, “I feel I am doing well in my life” were proved to be positively related to wellbeing, with a 2.2%, 4.2% and 3.6% increase of the wellbeing scale per increase of one point on a 5-point rating scale, respectively; “My life has a sense of routine” was proved to be negatively related to wellbeing, with 2.1% decrease of the wellbeing scale per increase of one point on a 5-point rating scale. Of the seven housing environment variables tested, only distance to a busy road, noise annoyance and range hoods in kitchen were significant. The wellbeing levels of respondents having no smoke lampblack machine or range hood in their kitchen were on average 4.8% lower than others. Regarding the noise annoyance, the less frequent noise annoyance was associated with higher wellbeing but living further away from a busy road was associated with significantly lower wellbeing. This may be the result of conflicts between the many factors that impact wellbeing. Living near a busy road may make access to jobs and a social life easier, both of which enhance wellbeing. Alternatively it might reflect citizen’s acceptance of background traffic noise—they are simply accustomed to it.

## 4. Discussion

### 4.1. Comparisons with Other Studies

Results of regression modelling suggested that subjective wellbeing was associated with age, satisfaction, ontological security and the housing environment. Generally, this study has common findings with other similar studies of wellbeing. For example, the middle aged respondents had lowest wellbeing, has also been found in other studies [58]. Other studies also found that respondents who were healthy and had good social relationships had higher levels of wellbeing [59]. Higher levels of wellbeing were also found among respondents who felt safe and led lives structured by routine, which help improve mental health [60].

There are also some different findings from other studies. Firstly, many previous studies have proved that socioeconomic status was significantly associated with wellbeing [61,62] while our measure of socioeconomic status was not significant. However, it should be noted that there were indications that status was associated with wellbeing in this study: respondents with higher levels of wellbeing could also afford a range hood, and a home with no traffic noise; such respondents saw themselves as someone who was doing well and who other people would like to be. Thus our SES may not have been significant because it was accounted for by other variables. For example, we only considered the ownership of their home as the measurement of SES but not the location and neighborhood quality of their house. Obviously, the SES of respondents owning their home in a good community will be significantly different from those owning their home in a troubled community. But using our measurement of SES, they are the same. Alternatively we perhaps should have considered more factors in our measure of SES in the future. Secondly, respondents’ satisfaction with air quality and their attitude and response to air pollution issues haven’t been proved to significantly affect wellbeing in this study, which was quite different from conclusions in previous studies [56]. This may be explained by the fact that quite good air quality during the survey period made people less concerned about this factor. Thirdly, unlike other studies [63] we did not find that distance from green space was associated with wellbeing. This might be because the usage and quality of the green space were not measured [64]. Finally, it should be noted that previous studies have provided quantitative or qualitative evidences for the relationships between wellbeing and factors like the response to stress and change [46,47], the freedom of doing what they like [46,47], perspective on future air quality [14,56], mental and physical health status [59,60], but in this study maybe the sample size limited them from being statistically significant.

### 4.2. Climate Change Policy Analysis

**Figure 1:**
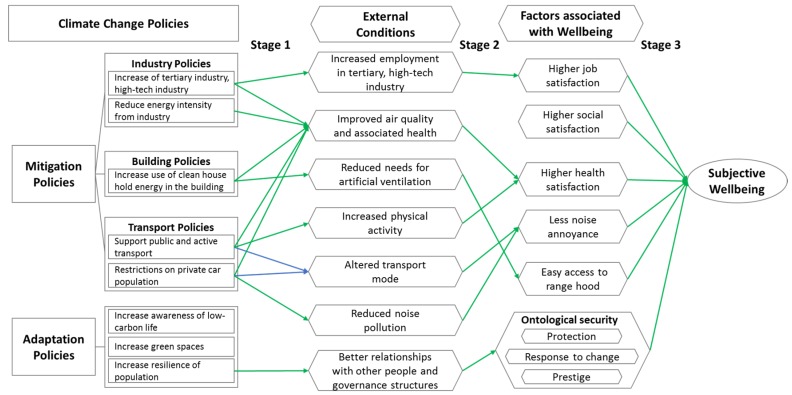
Putative pathways through which climate change policies might connect with wellbeing. Green lines indicate the positive relationships; Blue lines indicate neutral relationships.

By matching the conditions changed by climate change policies with factors significantly associated with wellbeing, Figure 1 showed the putative pathways through which climate change policies promote or conflict with subjective wellbeing. The pathways were expressed as chains of connections, which can be divided into three stages. Stage 1 described how policies directly or indirectly affected external conditions; Stage 2 described the connections between these external conditions and wellbeing associated factors suggested by the regression model; Stage 3 described how these wellbeing associated factors influenced subjective wellbeing (see also Table 5). Only when all stages are connected can the valid pathways through which climate change policies promote or conflict with subjective wellbeing form. Otherwise, climate change policies are considered to have no impacts on wellbeing.

Based on Figure 1, we found that none of existing climate change strategies in Suzhou conflicted with subjective wellbeing. Four strategies, namely industry policies like the increase of tertiary industry and high-tech industry and reduced energy intensity, increased use of renewable energy in buildings, transport policies, and increasing people’s resilience, tended to bring co-benefits for population wellbeing. The specific pathways were described as follows.

Increased tertiary and high tech industries indicates that there will be a shift from traditional secondary industry to tertiary and high tech industries in Suzhou. This shift may provide more available jobs in tertiary and high tech industries. Jobs in tertiary and high tech industries are significantly high-paid than that in traditional secondary industries [65], and pay is the most important contributor to higher job satisfaction. Higher levels of job satisfaction were significantly associated with higher wellbeing in our study. Additionally, both reduced energy intensity and replacing high emission industry with tertiary and high tech industry [66,67] will result in reduced air pollution and hence associated health improvement. Higher levels of satisfaction with health was strongly related to increased wellbeing. Thus, increasing tertiary industry and high–tech industry may result in higher levels of wellbeing in Suzhou. 

The reason why respondents owning artificial ventilation systems like range hoods in their kitchen reported significantly higher wellbeing was probably because they would not suffer the smoke and smell generated by cooking [30]. Substituting traditional household energy with renewable energy in buildings can play the same role as range hoods on oil fumes and smell removal and reduce residents’ needs for artificial ventilation [68]. Therefore, with the increase of renewable energy in buildings, residents will gain higher wellbeing.

Traffic policies like encouraging public transport and restricting car use may promote wellbeing via different pathways. Firstly, these policies will reduce the health damage caused by traffic related air pollutants and hence increased resident’s satisfaction with their health, which significantly affects wellbeing. Moreover, it might improve wellbeing as streets might become less noisy and the general level of noise annoyance at home are reduced. It should be noted here that though the reduced car use here also means that the roads will become less busy and the relative distance to busy road will be increased, the wellbeing will not be altered through this pathway. Because the things make people living near to busy road happier are easier access to jobs and a social life rather than the distances itself. In this case, however, the only thing changed is the distance. In addition, according to our results, the changes of transport mode will not affect wellbeing, which are based on the assumptions that if car users were forced to abandon their car and take public transport, their wellbeing would become equal to the level of original users of public transport. Actually, losing a privilege such as total freedom of private vehicle use, is likely to reduce wellbeing in the short term. Nevertheless there are benefits of good public transport and active transport, such as cleaner air and increased physical activity. So in the long term once people have become accustomed to restricted car use, there may not be much difference in wellbeing. Unfortunately, the cross sectional data in this study could not catch this dynamic process. In the future, follow-up studies should be performed to consider these longitudinal impacts of the policies on wellbeing.

Resilience involves being able to adapt and/or recover from disturbances which are likely to become more common due to climate change [69]. Resilient people can be described as having “ontological security”, a sense of continuity which can be developed through feeling valued, feeling protected and feeling in control [47]. The results of the survey implied that higher levels of wellbeing were found among respondents who were doing well and had a life that other people would value and feel safe. Additionally those with higher levels of wellbeing did not lead lives of dull routine. Thus the authorities in Suzhou should value all the people in the city to increase their resilience. Actually, they already are doing so. According to the document of “Low carbon development planning of Suzhou (2011–2020)” [25], the policy of “increase resilience” in Suzhou includes two important aspects. At city or community level, the first is to improve the ability of existing public facilities (drinking water supply system, energy supply system, transport and communication facilities) to adapt to extreme events caused by climate change; the second is to strength the constructions of climate–related public facilities, including urban flood control facilities, drainage system, water–saving irrigation system meteorological disaster alarming information issue system, vector borne disease prevention and control system; the third is to provide easy access to consultation service, health care on climate-related disease; the forth is to improve residents’ awareness on climate change related issues via lectures, hotline, television, radio, newspapers and Internet. At individual level, residents are encouraged to learn more climate related knowledge and adopt personal protective measures like air conditioning systems, sunscreen products, warming or cooling products *etc.* Overall, all these measures strengthen residents’ relationship with community neighbours and governance structure and make them feel protected and valued, which can help them respond to climate change issues better.

### 4.3. Uncertainties and Limitations

Uncertainties and limitations of this study mainly came from the study design, the content and format of questionnaire. We give a detailed discussion in the following paragraphs.

The first and major concern of this study is the use of only cross sectional data. Specifically, the cross sectional data allow us to examine the association between wellbeing and external conditions but not identify valid and reliable “ratios” of wellbeing status associated with certain levels of exposure. It may be the case that high levels of wellbeing caused the respondents to report high levels of satisfaction and low levels of noise annoyance for example rather than these elements developing wellbeing. Moreover, the cross sectional data provide little information on temporal dynamics of the association which should be better understood to support the policy making. In this study some factors affected by urban climate change policies, like the change of transport mode, satisfaction with air quality and environmental opinions, had no significant impact on wellbeing but this might be because associations were perhaps just city-specific or time-specific. It is likely that as time goes on, the impacts of environmental factors on wellbeing might become much more significant and stronger due to sharply increased risk perception of environmental problems. An ideal way to assess the wellbeing implications of climate change policies is to evaluate differences in wellbeing between pre-policy and post-policy based on a longitudinal dataset. We plan to use the cross sectional survey data to provide an initial baseline from which a follow-up study can be performed to consider impacts of the policies on wellbeing in the future.

The second problem challenging this study is the issues regarding questionnaire design. As we know, there are many factors affecting the subjective wellbeing, some of which are even unknown. It is impossible to take all factors into consideration in this study. For example, questions like how often respondents spend in green spaces, outdoors, on public transport, cooking at home, and subjective ratings of air quality (indoor/outdoor) haven’t been asked. In general, however, the omitted factors would significantly alter the wellbeing findings because we identified the most important factors affecting wellbeing and put them into the questionnaire based on a relatively comprehensive literature review before the design of the questionnaire. In addition, the specific way questions are asked will affect the responses and the results. Actually, we have tried to minimize the effects of questions on the response and the results in this study. Specifically, we adopted the widely-used and well-tested scales or questions to measure subjective concepts such as the WHO–5 scale to measure wellbeing. In terms of resilience, there are various measures that have measured for example equanimity, perseverance, self-reliance, meaningfulness and impulse control although most are still under development [70]. In this study, we chose to introduce the concept of ontological security to the study of the impact of climate change on resilience because of its central importance to mental health and wellbeing and theoretical underpinning of negative and positive responses to external change. Regarding the external conditions, the questions were asked as objectively as possible to reduce the disturbances of specific operationalization on participants’ responses.

### 4.4. Policy Implications

This study represents a first attempt at screening the climate change policies which potentially enhance or undermine subjective wellbeing. Though the cross sectional data we used cannot make causal attributions, it still provided important policy implications for other cities in China as well as Suzhou.

Firstly, although the main objective of the interventions implemented by the cities targets the reduction of greenhouse gas emission, the impacts on wellbeing should be included in the cost effectiveness analysis (CEA) of urban climate change mitigation and adaptation policies. Taking wellbeing into consideration is expected to significantly change the policy making process. Given that the policies are intended to reduce greenhouse gas emissions rather than improve public health, large health and wellbeing gains are not expected and even small health and wellbeing improvements should be evaluated positively. Fortunately, according to our study, many strategies to cope with climate change are significantly associated with positive wellbeing effects rather than conflicts.

Secondly, the dynamic characteristics of associations between wellbeing and its potential predictors should be better understood to support the policy making. In this study some factors affected by urban climate change policies, like air quality and environmental opinions, had no significant impacts on wellbeing but this might because associations were perhaps just city-specific or time-specific. It is likely that as time goes on, the impacts of environmental factors on wellbeing might become much more significant and stronger due to sharply increased perceived risk of environmental problems. Therefore, further longitudinal studies are required to develop our understanding on this question in the future.

Finally, there may be a mismatch between climate change policy beneficiaries and victims in terms of wellbeing. For example, the implementation of industrial structural adjustment may result in people with low SES sacrificing income from their low-end jobs for a healthy living environment and hence higher wellbeing for people in all groups. Restrictions on private car use might reduce noise and hence bring wellbeing benefits for residents living near to a busy road, but it sacrifices the convenience of car owners usually with high SES. Thus, inequalities of the interventions should be seriously considered before the implementation.

## 5. Conclusions

In this study, exploratory wellbeing impact assessments of climate change mitigation and adaptation policies in Suzhou are calculated through the described methodology that makes use of data from a local survey and official documents. The main findings are concluded as follows. Firstly, the statistical analysis indicated that eleven variables, including age, satisfaction with job, health and social life, ontological security variables of “other people would like a life like mine”, “I feel safe”, “I feel I am doing well in my life” and “My life has a sense of routine”, distance to a busy road, noise annoyance and range hoods in kitchen, were significantly associated with people’s wellbeing in Suzhou. On the basis of this, policy analysis implies that climate change policies for promoting tertiary and high-tech industry, renewable energy in buildings, and limitation of total vehicle population may have positive effects on wellbeing. There is no evidence supporting that change of transport mode and increase of green spaces would reduce or increase wellbeing. Helping citizens to have a satisfying social life, to feel safe, and to have self–worth and resilience could also improve their wellbeing. Lastly, though climate change interventions are found to be significantly associated with positive wellbeing benefits in Suzhou, the potential conflicts of interventions with health and wellbeing should always be considered in the framework of policy making. We also suggest that further studies for dynamic wellbeing effects and inequalities resulting from urban interventions should be conducted.

## Figures and Tables

**Table 1:** The list of climate change mitigation and adaptation policies in Suzhou.

Policy Category	Specific Measures	External Conditions Affected
Mitigation policy	Increase of tertiary industry, high-tech industry	Employment rate, quality and salary [27]
		Air quality and related health [2,28]
	Reduce energy intensity from industry	Air quality and related health [2,28]
	Increase use of clean household energy in the building	Indoor air quality [29]
		The use of artificial ventilation system like the air conditioner and kitchen range hoods [30]
		Housing quality [31]
	Support public and active transport	Transport mode [32]
		Air quality [33,34]
		Physical activity and health [35,36,37]
	Restrict private car population	Transport mode [26,32]
		Air quality [33,34]
		Noise pollution [33,34]
	Increase awareness of low-carbon life	Air quality [33,34]
		Environmental opinions [38]
Adaptation policy	Increase green spaces	Quantity/quality of green space [39]
	Increase resilience of population	Relationships with other people and governance structures [40]

**Table 2:** Descriptions of the independent variables.

Group	Variables	Types	Measures
**Personal**	Age	Categorical	Four bins: ≤22, 23–39, 40–59, and ≥60
	Gender	Categorical	Female *vs.* Male
	Socioeconomic status (SES)	Categorical	Three bins: High, Mid, and Low SES
**Health**	Physical conditions	Categorical	Has medical condition ^**a**^ *vs.* No medical condition
	Depression	Categorical	Depressed *vs.* Not depressed
	Smoking	Categorical	Smoker *vs.* Non–smoker
**Satisfaction**	Satisfaction with job, family life, apartment, neighbor, social life, air quality and health	Continuous	Measured on a 5 point Likert scale with responses ranging from highly unsatisfied to highly satisfied
**Ontological security**	“I enjoy a challenge”, “I can deal with stress”, “I’m frightened of change”, “I can do what I want, when I want”, “Most people would like a life like mine”, “I feel in control”, “I feel safe”, “I worry about things going wrong”, “I feel I’m doing well in life“, “My life has a sense of routine”	Continuous	Measured on a 5 point Likert scale with responses ranging from strongly agree to strongly disagree
**Housing environment**	The use of heating/cooling	Categorical	Three bins: No devices/never use, partly use, and full use
	Distance to green space	Categorical	Three bins: Less than 10-min walk, 10-min to 30-min walk, and More than 30-min
	Near to busy road	Categorical	Yes *vs.* No
	Annoyed by the noise when you are at home	Categorical	Three bins: At no time, Less than half of the time, and More than half of the time
	Main household fuel type	Categorical	Five bins: Coal/wood, Liquefied gas, Electricity, Natural gas, and other
	The use of range hood in kitchen	Categorical	Three bins: Yes, No, and Other
	Exchange small favors	Categorical	Four bins: Never, Once a week, 2–4 time a week, more than 4 times a week
**Other**	Environmental opinions (seven variables included)	Continuous	Measured on a 5 point Likert scale with responses ranging from strongly agree to strongly disagree with the statements
	Main mode of transport in summer	Categorical	Six bins: Motorbike, Car, Bike, Walk, Public transport and Other

^**a**^ Conditions include cerebral infarction, coronary heart disease, myocardial infarction, other heart diseases, chronic obstructive pulmonary disease (COPD), chronic bronchitis, asthma, and lung cancer.

**Table 3:** The conception of ontological security.

Concept	Items
**Protection**	I can deal with stress; I feel safe
**Control**	I feel in control; I can do what I want, when I want;
**Prestige**	Most people would like a life like mine; I feel I’m doing well in life
**Response to change**	My life has a sense of routine; I worry about things going wrong (reversed); I enjoy a challenge, I’m frightened of change (reversed)

**Table 4:** Descriptive statistics of wellbeing data in Suzhou.

Descriptive Statistics		WHO–5 Scale 0–100
**N**	**Valid**	763
	**Missing**	12
**Mean**		55.51
**Skewness**		−0.446
**Std. Error of Skewness**		0.089
**Minimum**		0
**Maximum**		100

**Table 5:** The results of generalized estimation equation modelling.

Design Variables		N	%	b (95% CI)
SES	Low SES (unemployed or basic schooling or low household income plus no high SES characteristics)	84	11.7	−2.4 (−6.8 to 2.1)
	Mid SES	465	64.9	−0.4 (−3.2 to 2.4)
	High SES (lives in owner occupied house or high household income plus no low SES characteristics)	167	23.3	0 ^**a**^
Age	≤22	224	31.3	8.8 (3.7 to 14.0)
	23–39	385	53.8	4.6 (−0.2 to 9.4)
	40–59	67	9.4	0 ^**a**^
	60+	40	5.6	4.1 (−2.9 to 11.1)
Gender	Male	326	45.5	−0.9 (−3.2 to 1.4)
	Female	390	54.5	0 ^**a**^
**Added variables**				
Satisfaction	Satisfied with job			2.2 (0.9 to 3.5)
	Satisfied with social			1.8 (0.5 to 3.2)
	Satisfied with health			4.6 (2.7 to 6.5)
Ontological security	Most people would like a life like mine			2.2 (0.6 to 3.8)
	I feel safe			4.2 (2.8 to 5.5)
	I feel I am doing well in life			3.6 (1.6 to 5.7)
	My life has a sense of routine			−2.1 (−3.6 to −0.7)
Housing environment	Not near to busy road	253	35.3	−2.9 (−5.2 to −0.5)
	Near to busy road	463	64.7	0 ^**a**^
	Annoyed by the noise when you are at home at no time	73	10.2	4.9 (0.7 to 9.0)
	Annoyed by the noise when you are at home at less than half of the time	506	70.7	2.9 (−0.1 to 5.8)
	Annoyed by noise when at home at more than half of the time	137	19.1	0 ^**a**^
	No smoke lampblack machine/range hood in kitchen	89	12.4	−4.8 (−8.5 to −1.0)
	Other	40	5.6	−3.7 (−9.2 to 1.8)
	Smoke lampblack machine/range hood in kitchen	587	82.0	0 ^**a**^

Notes: For “SES”, “Satisfaction”, “Ontological security”, “noise”, missing values are recoded as midpoint; for “range hood”, recoded as “other”; for “age”, recoded as “23–39”; 0 ^**a**^ indicates the reference group to which other bins of the variable are compared.

## Supplementary Materials

The following are available online at www.mdpi.com/1660-4601/13/3/344, Table S1: The details of eight sample sites, Table S2: Demographic characteristic of respondents, Table S3: How satisfied are you with the following issues, Table S4: Ontological security: Below are some opinions people might have about themselves. How strongly do you agree or disagree with each one, Table S5: Home environment variables, Table S6: Transport model, Table S7: Below are some statements about environment. How strongly do you agree or disagree with each one, Table S8: Health variables, Table S9: Design variables.
