# Supplementary material for: Do Climate Change Policies Promote or Conflict with Subjective Wellbeing: A Case Study of Suzhou, China
Source: Int J Environ Res Public Health. 2016 Mar 21;13(3):344. doi: 10.3390/ijerph13030344 (PMC4809007; doi:10.3390/ijerph13030344)
Supplement: Supplementary file 1 [file ijerph-13-00344-s001.pdf]

Supplementary Materials: Do Climate Change Policies Promote or Conflict with Subjective Wellbeing: A Case Study of Suzhou, China

Miaomiao Liu, Yining Huang, Rosemary Hiscock, Qin Li, Jun Bi, Patrick L. Kinney and Clive E. Sabel

Table S1. The details of eight sample sites.

Sites ID	Name	Location	Number of Samples	Description of Sites
A	Guanqian Street-1	Gusu	109	Guanqian Street is the bustling downtown of Suzhou and is frequented by people from all districts. Due to the large potential typical samples with all ages and large area itself, we set two sites in Guanqian Street.
B	Guanqian Street-2	Gusu	71	
C	Auchan Supermarket	SIP	121	Many people living in Suzhou Industrial Park (SIP), visit Auchan Supermarket.
D	Suxiu Road	SIP	101	Suxiu Road, is the location of many banks in SIP and visited by numbers of local people.
E	Suzhou Railway Station	Gusu	71	Suzhou Railway Station has lots of foot traffic from all districts of Suzhou.
F	Shantang Street	Gusu	117	Shantang Street is acclaimed as "the number one street in Gusu" and is located in the historical zone in Suzhou. It is mostly frequented by local people.
G	Shilu Street	Gusu	119	Shilu is a pedestrian street with hundreds of historic famous shops along it, and is visited by a large number of people living in Gusu.
H	Meiluo Square	Gaoxin	76	Gaoxin district in western Suzhou is another industrial development zone. Meiluo Square is visited by large numbers of people working in High-Tech industry.

Table S2. Demographic characteristic of respondents.

Demographic Characteristic Variables	Survey n = 775 (%)	Population of Suzhou n = 6.4 Million (%)
**Sex**		
Female	54	51
Male	46	49
**Age in years**		
≤39	85	45
≥40	15	55
**Education**		
primary or below	1	32
Junior school	8	32
Senior or Secondary specialized school	23	18
Junior college	24	10
Bachelor	40	7
Master or above	4	1
**Household monthly income**		
<2000 RMB	4	40
2000–4999 RMB	31	51
≥5000 RMB	65	9

Table S3. How satisfied are you with the following issues?

Observations		Your Current Job		Your Family Life		Your Apartment		Your Neighbors		Your Social Life		Air Quality		Your Health	
		N	%	N	%	N	%	N	%	N	%	N	%	N	%
Valid	1 highly unsatisfied	51	6.6	20	2.6	32	4.1	29	3.7	26	3.4	79	10.2	20	2.6
	2	84	10.8	53	6.8	94	12.1	77	9.9	91	11.7	197	25.4	281	36.3
	3	281	36.3	161	20.8	204	26.3	239	30.8	303	39.1	306	39.5	379	48.9
	4	213	27.5	276	35.6	261	33.7	254	32.8	237	30.6	144	18.6	84	10.8
	5 highly satisfied	115	14.8	255	32.9	178	23	165	21.3	109	14.1	42	5.4	-	-
	total	744	96	765	98.7	769	99.2	764	98.6	766	98.8	768	99.1	764	98.6
Missing		31	4	10	1.3	6	0.8	11	1.4	9	1.2	7	0.9	11	1.4
**Total**		**775**	**100**	**775**	**100**	**775**	**100**	**775**	**100**	**775**	**100**	**775**	**100**	**775**	**100**
**Median Wellbeing**															
1 highly unsatisfied			46.0		60.0		54.0		60.0		56.0		52.0		34.0
2			48.0		44.0		44.0		52.0		50.0		56.0		52.0
3			52.0		52.0		52.0		52.0		52.0		56.0		60.0
4			64.0		56.0		60.0		60.0		64.0		60.0		68.0
5 highly satisfied			68.0		64.0		64.0		64.0		64.0		64.0		-
**total**			**56.0**		**56.0**		**56.0**		**56.0**		**56.0**		**56.0**		**56.0**
**Spearman's rho**			**0.319 ****		**0.279 ** **		**0.256 ** **		**0.202 ** **		**0.303 ** **		**0.163 ** **		**0.276 ** **
p			0.000		0.000		0.000		0.000		0.000		0.000		0.000
n			733		754		758		754		756		757		754
**GLM controlling for design variables**															
**b**		**2.2 ****		**0.6**		**0.4**		**−0.6**		**1.8 ****		**0.8**		**4.6 ****	
**95% CI**		**(0.9 to 3.5)**		**(−0.8 to 1.9)**		**(−0.9 to−1.7)**		**(−2.0 to 0.7)**		**(0.5 to 3.2)**		**(-0.4 to 2.0)**		**(2.7 to 6.5)**	

** $p < 0.05$; missing cases codes as midpoint 3; 0^a is the reference group that other levels of the factor are compared to.

Table S4. Ontological security: Below are some opinions people might have about themselves. How strongly do you agree or disagree with each one?

**Observations**		**I Enjoy a Challenge**		**I Can Deal with Stress**		**I'm Frightened of Change**		**I Can Do what I Want, When I Want**		**Most People Would Like a Life Like Mine**	
			**%**	**N**	**%**	**N**	**%**	**N**	**%**	**N**	**%**
Valid	1 strongly disagree	4	0.5	3	0.4	27	3.5	17	2.2	20	2.6
	2	44	5.7	39	5	181	23.4	241	31.1	178	23
	3	248	32	187	24.1	299	38.6	267	34.5	364	47
	4	337	43.5	446	57.5	201	25.9	178	23	162	20.9
	5 strongly agree	137	17.7	95	12.3	52	6.7	60	7.7	40	5.2
	total	770	99.4	770	99.4	760	98.1	763	98.5	764	98.6
Missing		5	0.6	5	0.6	15	1.9	12	1.5	11	1.4
**Total**		**775**	**100**	**775**	**100**	**775**	**100**	**775**	**100**	**775**	**100**
**Mean wellbeing**											
1 strongly disagree			60		36		64		48		26
2			50		40		60		52		48
3			56		52		56		60		60
4			60		60		56		64		64
5 strongly agree			60		60		52		64		64
**total**			**56**		**56**		**56**		**56**		**56**
**Spearman's rho**			**0.130 ****		**0.176 ****		**−0.085 ***		**0.228 ****		**0.303 ****
p			0		0		0.02		0		0
n			760		760		751		753		754
**GLM controlling for design variables**											
**b**		**−0.4**		**0.7**		**0.6**		**0.2**		**2.2 ****	
**95% CI**		**(−2.0 to 1.2)**		**(−1.2 to 2.6)**		**(−0.6 to 1.9)**		**(−1.2 to 1.6)**		**(0.9 to 3.5)**	
**Observations**		**I Feel in Control**		**I Feel Safe**		**I Worry about Things Going Wrong**		**I Feel I'm Doing Well in Life**		**My Life Has a Sense of Routine**	
		**N**	**%**	**N**	**%**	**N**	**%**	**N**	**%**	**N**	**%**
Valid	1 strongly disagree	13	1.7	16	2.1	37	4.8	2	0.3	15	1.9
	2	157	20.3	119	15.4	263	33.9	42	5.4	92	11.9
	3	274	35.4	223	28.8	235	30.3	193	24.9	267	34.5
	4	276	35.6	344	44.4	189	24.4	454	58.6	342	44.1
	5 strongly agree	43	5.5	59	7.6	38	4.9	65	8.4	49	6.3
	total	763	98.5	761	98.2	762	98.3	756	97.5	765	98.7

Table S4. *Cont.*

Observations	I Feel in Control		I Feel Safe		I Worry about Things Going Wrong		I Feel I'm Doing Well in Life		My Life Has a Sense of Routine	
	**N**	**%**	**N**	**%**	**N**	**%**	**N**	**%**	**N**	**%**
Missing	12	1.5	14	1.8	13	1.7	19	2.5	10	1.3
**Total**	**775**	**100**	**775**	**100**	**775**	**100**	**775**	**100**	**775**	**100**
**Mean wellbeing**										
1 strongly disagree		60		36		64		34		64
2		50		48		60		40		62
3		56		56		56		48		60
4		64		60		56		60		56
5 strongly agree		72		76		50		72		48
**total**		**56**		**56**		**56**		**56**		**56**
**Spearman's rho**		**0.264** **		**0.333** **		**−0.117** **		**0.353** **		**−0.160** **
p		0		0		0.001		0		0
n		754		751		752		746		754
**GLM controlling for design variables**										
**b**	**0.5**		**4.2** **		**0.6**		**3.6** **		**−2.1** **	
**95% CI**	**(−1.1 to 2.1)**		**(2.8 to 5.5)**		**(−0.6 to 1.7)**		**(1.6 to 5.7)**		**(−3.6 to −0.7)**	

* $p < 0.10$; ** $p < 0.05$; missing cases are coded as midpoint 3; 0[a] is the reference group that other levels of the factor are compared to; Scale reversed for "I'm frightened of change", "I worry about things going wrong".

Table S5. Home environment variables.

**Variables**	**N**	**%**	**Median Wellbeing**	**GLM b (95% CI)**
Access to heating and cooling devices in your house			***p*** **= 0.002**	
No devices/ never use	65	8.4	48.0	0.1 (−3.8 to 4.0)
partly use	85	11.0	56.0	−3.0 (−6.7 to 0.7)
full use	597	77.0	60.0	0 [a]
total	747	96.4	56.0	
System	28	3.6		
Total	775	100.0		
Distance to green space [a]			***p*** **= 0.004**	
less than 10-min walk	403	52.0	60.0	0.8 (−2.7 to 4.4)
10-min to 30-min walk	243	31.4	56.0	−0.5 (−4.0 to 3.1)
more than 30-min walk	114	14.7	56.0	0 [a]
total	760	98.1	56.0	
System	15	1.9		
Total	775	100.0		
Near to busy road			***p*** **= 0.001**	
No	267	34.5	56.0	−2.9 ** (−5.2 to −0.6)
Yes	498	64.3	60.0	0 [a]
total	765	98.7	56.0	
System	10	1.3		
Total	775	100.0		
Annoyed by the noise when you are at home [a]			***p*** **= 0.005**	
At no time	76	9.8	64.0	5.3 ** (1.1 to 9.4)
less than half of the time	534	68.9	60.0	2.8 (−0.1 to 5.7)
more than half of the time	154	19.9	52.0	0 [a]
total	764	98.6	56.0	
System	11	1.4		
Total	775	100.0		

Table S5. *Cont.*

Variables	N	%	Median Wellbeing	GLM b (95% CI)
Main household fuel type [b]			p **= 0.110**	
Coal & wood	32	4.1	58.0	−6.1 (−11.9 to −0.3)
Liquefied gas	173	22.3	56.0	0.1 (−2.7 to 2.8)
Electricity	105	13.5	56.0	−2.7 (−6.6 to 1.2)
Other	22	2.8	56.0	−2.2 (−7.2 to 2.9)
Natural gas	413	53.3	60.0	0 [a]
total	745	96.1	56.0	
System	30	3.9		
Total	775	100.0		
Is there smoke lampblack machine/range hood in your kitchen?			p **= 0.010**	
No	101	13.0	52.0	−4.6 ** (−8.3 to −0.9)
Other	56	7.2	60.0	−4.0 (−9.5 to 1.4)
Yes	618	79.7	60.0	0 [a]
Total	775	100.0	56.0	
Frequency of exchanging small favors with your neighbor [c]			p **= 0.636**	
Never	227	29.3	60.0	0.2 (−3.2 to 3.7)
Once a week	52	6.7	56.0	−0.8 (−5.8 to 4.2)
2–4 times in a week	210	27.1	60.0	1.1 (−2.0 to 4.2)
more than 4 times in a week	164	21.2	56.0	0 [a]
total	653	84.3	56.0	
System	122	15.7		
Total	775	100.0		

** $p < 0.05$; [a] missing values are recoded as midpoint 2; [b] missing value are recoded as "other"; [c] missing values are recoded as the median; 0 [a] is the reference group that other levels of the factor are compared to.

Table S6. Transport model.

Transport Mode in Summer	N	%	Median Wellbeing	GLM b (95% CI)
**I make most of my journeys by:**			***p* = 0.580**	
Motorbike	29	3.7	60	2.8 (−2.6 to 8.1)
Car	80	10.3	56	−1.8 (−5.5 to 1.9)
Bike	43	5.5	56	2.1 (−2.5 to 6.7)
Walk	44	5.7	64	1.2 (−4.4 to 6.9)
Other [a]	363	46.8	60	0.1 (−2.6 to 2.8)
Public transport	216	27.9	56	0 [a]
Total	775	100.0		

[a] missing cases are coded as "other"; 0 [a] is the reference group that other levels of the factor are compared to; Because questionnaire survey was conducted in the summer, winter journeys were not included in the multivariate modeling.

Table S7. Below are some statements about environment. How strongly do you agree or disagree with each one?

**Observations**	**Air Pollution Could be Ignored in the Past 2 Weeks**		**Air Pollution Has Negative Impacts on Physical Health**		**Air Pollution Has Negative Impacts on Happiness**		**Sacrifice some Air Quality to the Faster Economic Development**		**Suzhou's Air Quality Will be Improved in Near Future**		**Air Quality Improvement Will Benefit Your Physical Health**		**Air Quality Improvement Will Benefit Your Happiness**	
	**N**	**%**	**N**	**%**	**N**	**%**	**N**	**%**	**N**	**%**	**N**	**%**	**N**	**%**
1 strongly disagree	58	7.5	23	3.0	26	3.4	469	60.5	36	4.6	13	1.7	6	0.8
2	169	21.8	81	10.5	101	13.0	139	17.9	230	29.7	12	1.5	25	3.2
3	254	32.8	180	23.2	196	25.3	63	8.1	90	11.6	81	10.5	101	13.0
4	120	15.5	208	26.8	215	27.7	45	5.8	373	48.1	175	22.6	213	27.5
5 strongly agree	43	5.5	205	26.5	169	21.8	24	3.1	46	5.9	442	57.0	384	49.5
total	644	83.1	697	89.9	707	91.2	740	95.5	775	100.0	723	93.3	729	94.1
Missing	131	16.9	78	10.1	68	8.8	35	4.5	0	0.0	52	6.7	46	5.9
**Total**	**775**	**100.0**	**775**	**100.0**	**775**	**100.0**	**775**	**100.0**	**775**	**100.0**	**775**	**100.0**	**775**	**100.0**
**Median wellbeing**														
1 strongly disagree		56		68		66		56		64		46		52
2		56		60		60		56		60		64		50
3		56		56		56		56		60		56		56
4		56		60		56		60		56		56		56
5 strongly agree		64		56		60		56		56		60		60
**total**		**56**		**56**		**56**		**56**		**56**		**56**		**56**
**Spearman's rho**		**0.093 ***		**−0.075**		**−0.074**		**−0.019**		**−0.127 ****		**0.073**		**0.088 ***
p		0.019		0.050		0.052		0.603		0.000		0.050		0.018
n		635		689		699		730		763		716		721
**GLM controlling for design variables**														
**Sqrt(b)**	**0.2**		**0.2**		**0.2**		**−0.1**		**0.8**		**0.7**		**0.8**	
**95% CI**	**(−1.0 to 1.5)**		**(−1.0 to 1.3)**		**(−0.9 to 1.3)**		**(−1.2 to 1.0)**		**(−0.2 to 1.9)**		**(−0.5 to 2.0)**		**(−0.5 to 2.1)**	

* $p < 0.10$; ** p < 0.05; missing cases are coded as midpoint 3.

Table S8. Health variables.

Variables	N	%	Median Wellbeing	GLM b (95% CI)
***Physical medical condition* (stroke, COPD, heart, asthma, bronchitis, lung cancer in the past year diagnosed, inpatient, outpatient or attended ER)**			**p = 0.005**	
No	733	94.6	60	1.2 (−5.8 to 8.3)
Yes	36	4.6	44	0 [a]
total	769	99.2	56	
System	6	0.8		
Total	775	100.0		
Depression (past year diagnosed, inpatient, outpatient or attended ER			**p = 0.021**	
No	756	97.5	60	7.0 (−8.4 to 22.3)
Yes	6	0.8	26	0 [a]
total	762	98.3	56	
System	13	1.7		
Total	775	100.0		
Smoking [a]			**p = 0.090**	
other	130	16.8	60	6.1 (1.6 to 10.5)
nonsmoker	532	68.6	56	2.8 (−0.8 to 6.3)
smoker	113	14.6	56	0 [a]
Total	775	100.0	56	

[a] Smoking not included in final model due to large number of missing values; 0 [a] is the reference group that other levels of the factor are compared to.

Table S9. Design variables.

Design Variables	N	%	Median Wellbeing	GLM b (95% CI)
Age			***p* = 0.001**	
≤22	232	29.9	64	8.6 ** (3.4 to 13.8)
23–39	395	51.0	56	4.6 * (−0.3 to 9.4)
60+	45	5.8	56	4.2 (−2.8 to 11.3)
40–59	70	9.0	56	0[a]
total	742	95.7	56	
System	33	4.3		
Total	775	100.0		
Gender			***p* = 0.000**	
Male	343	44.3	56	−0.03 (−2.6 to 2.5)
Female	403	52.0	56	0 [a]
total	746	96.3	56	
System	29	3.7		
Total	775	100.0		
SES			***p* = 0.942**	
Low SES (unemployed or basic schooling or low household income plus no high SES characteristics)	87	11.2	56	−2.3 (−6.7 to 2.2)
Mid SES	293	37.8	60	−0.7 (−3.4 to 2.1)
High SES (lives in owner occupied house or high household income plus no low SES characteristics)	180	23.2	56	0 [a]
total	560	72.3	56	
System	215	27.7		
Total	775	100.0		

* $p < 0.10$; ** $p < 0.05$; 0 [a] is the reference group that other levels of the factor are compared to.

Appendix 1 Questionnaire

Dear participants:

Taking actions to address climate change has become the hotspot of the whole world, including Suzhou, where various measures have been implemented. These mitigation and adaptation actions can reduce greenhouse gas emissions, as well as affect your wellbeing via altering the surrounding external conditions. To better inform policy decisions, we designed a study to explore the impacts of these actions on wellbeing. To support this study, School of the Environment at Nanjing University is currently conducting an environment and health related questionnaire survey in Suzhou. The content of the questionnaire included five parts, namely wellbeing data, health data, living conditions, time activity diary and personal information.

You can participate our survey anonymously. All the information you provide will be kept strictly confidential and will be used only for this study. We promise no influence on your family or work. If you don't allow us to use your answers in our research or have any questions about the survey, please directly tell our volunteers or contact with the coordinating group:

Tel: 025-89680543
Fax: 025-89680533
E-mail: memo0904@sina.com
Address: School of the Environment, Nanjing University
Room B609, Qian Pansheng Building.
Nanjing 210023, China

Thank you for your support!

School of the Environment,
Nanjing University
August 2013

Part 1 Wellbeing Information

In the next five questions, we ask for your wellbeing in the last two weeks. Please let us know how much you agree with the statements.

Please tick ONE box for EACH statement.

1. Over the last two weeks, I have felt cheerful and in good spirit______
□ All of the time □ Most of the time □ More than half of the time
□ Less than half of the time □ Some of the time □ At no time
2. Over the last two weeks, I have felt calm and relaxed______
□ All of the time □ Most of the time □ More than half of the time
□ Less than half of the time □ Some of the time □ At no time
3. Over the last two weeks, I have felt active and vigorous______
□ All of the time □ Most of the time □ More than half of the time
□ Less than half of the time □ Some of the time □ At no time
4. Over the last two weeks, I woke up feeling fresh and rested______
□ All of the time □ Most of the time □ More than half of the time
□ Less than half of the time □ Some of the time □ At no time
5. Over the last two weeks, my daily life has been filled with things that interest me______
□ All of the time □ Most of the time □ More than half of the time
□ Less than half of the time □ Some of the time □ At no time

Part 2 Life Satisfaction

6. How satisfied are you with your life components? Please choose a number between 1 and 5 where 1 is *Not satisfied* at all and 5 is *Completely satisfied*.

Please tick ONE blank for EACH component.

**Life components**	**1**	**2**	**3**	**4**	**5**
Work situation					
Family life					
Housing conditions					
Neighbor					
Social life					
Air quality					

Part 3 Ontological Security

7. Below are some opinions that people might have about themselves. How strongly do you agree or disagree with each one? Please choose a number between 1 and 5 where 1 is *Strongly agree* and 5 is *Strongly disagree*.

Please tick ONE blank for EACH statement.

**Statements**	**1**	**2**	**3**	**4**	**5**
I enjoy a challenge					
I can deal with stress					
I'm frightened of change					
I can do what I want, when I want					
Most people would like a life like mine					
I feel in control					
I feel safe					
I worry about things going wrong					
I feel I'm doing well in life					
My life has a sense of routine					

Part 4 Health Information

8. How satisfied are you with your health? Please choose a number between 1 and 4 where 1 is Not satisfied at all and 4 is Completely satisfied.

□ 1 (Not satisfied at all) □ 2 □ 3 □4 (Completely satisfied)

9. Below are some opinions that people might have about themselves.

Please tick ONE blank for EACH disease

**In the last 12 months**	**Have you been attacked by the diseases below?**	Yes						No
	**Was it doctor diagnosis?**	Yes					No	
	**Have you been hospitalization for the diseases below?**	Emergency room		In hospital, as an inpatient		In hospital, as an outpatient		
		Yes	No	Yes	No	Yes	No	
**Diseases**	Cerebral infarction							
	Coronary heart disease							
	Myocardial infarction							
	Other heart diseases							
	Chronic obstructive pulmonary disease (COPD)							
	chronic bronchitis							

Asthma
Lung cancer
Depression

Part 5 Environment Opinions and Perceptions

10. In the next, we give seven statements about environment. Please let us know how much you agree with the statements. Please choose a number between 1 and 5 where 1 is *Strongly agree* and 5 is *Strongly disagree*.

Please tick ONE box for EACH statement

Statements	1	2	3	4	5
Air pollution could be ignored in the past two weeks					
Air pollution has negative impacts on my physical health					
Air pollution has negative impacts on my happiness					
It can be acceptable to sacrifice air quality for economic development					
Suzhou's air quality will be improved in near future					
Air quality improvement will benefit my physical health					
Air quality improvement will benefit my happiness					

Part 6 Housing and Environment

11. Housing ownership： □ Own □ Renting □ Others______
12. House type: □ House □ Apartment □ Others______
13. Main household cooking fuel:

□ Woods □ Coal □ Liquefied gas
□ Natural gas □ Electricity 14. □ Other______

15. Is there any smoke lampblack machine/range hoods in the kitchen? □ Yes □No
16. Is there any heating or cooling devices like air conditioner, Central heating in your house?
17. □ Yes □ No □ Other______

If yes, are they in working order? □ Yes □ Partly □ No

18. Is your house or apartment near to the busy road? □ Yes □ No
19. Main transport mode:

□ Walk □ Bike □ Motorcycle
□ Bus/Metro □ Taxi/Private car 20. □ Other______

21. Will you be annoyed by the noise when you are at home?

□ All of the time □ Most of the time □ More than half of the time
□ Less than half of the time □ Some of the time □ At no time

22. How many people are there in your neighborhood with whom you exchange small favors last two weeks?
An example would be leaving a key to let a repair man in.

Please WRITE the number of people in the boxes.

I exchange favors with [ ] people who live in my neighborhood in the last two weeks

23. How long does it take to go to public green space from your home?

□ Less than 10 min by walking □ 10–30 min by walking □ More than 30 min by walking

Part 7 Basic Information

24. Sex: ______ Age: ______ Occupation: ______
25. Education:

□ Primary School or below □ Mid school □ High school □ Secondary specialized school
□ Junior college □ Bachelor □ Master or above

26. Are you a smoker: □Yes □ No

If yes, how many cigarettes do you smoke one day _____

If no, are you an ex-smoker? □ Yes □ No

27. Family monthly income (RMB):

□ <500 □500–2000 □2000–5000 □5000–10,000
□10,000–20,000 □20,000–30,000 □30,000–50,000 □More than 50,000

© 2016 by the authors; licensee MDPI, Basel, Switzerland. This article is an open access article distributed under the terms and conditions of the Creative Commons by Attribution (CC-BY) license (http://creativecommons.org/licenses/by/4.0/).
